# CYR61 (CCN1) is a metastatic biomarker of gastric cardia adenocarcinoma

**DOI:** 10.18632/oncotarget.8845

**Published:** 2016-04-20

**Authors:** Jing Wei, Guanzhen Yu, Genbao Shao, Aiqin Sun, Miao Chen, Wannian Yang, Qiong Lin

**Affiliations:** ^1^ School of Medicine, Jiangsu University, Zhenjiang, Jiangsu, China; ^2^ Changzheng Hospital, Shanghai, China; ^3^ The Affiliated Hospital, Jiangsu University, Zhenjiang, Jiangsu, China

**Keywords:** gastric cardia adenocarcinoma, CYR61, prognostic biomarker, metastasis, cell migration

## Abstract

Gastric cardia adenocarcinoma (GCA) is the most aggressive subtype of gastric cancer with a high metastatic rate. In this report, we collected tumor tissue samples from 214 GCA cases and examined expression of CYR61, a target gene product of the Hippo-YAP/TAZ pathway, in the GCA tumors by immunohistochemical (IHC) staining using the tissue microarray assay (TMA). The results have shown that CYR61 is overexpressed in 44% of the GCA tumor samples. Expression of CYR61 is inversely correlated with cumulative survival of GCA patients (*p*<0.001) and significantly associated only with metastatic pathological categories (with N category, *p*=0.052; with TNM stage, *p*=0.001). Furthermore, knockdown of CYR61 in gastric cancer AGS cells impairs the cancer cell migration and invasion, suggesting a driver role of CYR61 in metastasis. Thus, our studies have established CYR61 as a metastatic biomarker for prediction of poor prognosis of GCA and provided a potential molecular target for anti-metastatic therapy of GCA.

## INTRODUCTION

Gastric cardia adenocarcinoma (GCA), the type II of gastroesophageal junction adenocarcinoma (GEJAC), is the most aggressive type of gastric carcinoma [1–3]. Clinical data indicate that the post-surgery recurrence rate of GCA is as high as 50% [4]. Metastasis, particularly the distant metastasis, is the major cause for poor prognosis and low survival rate of GCA [1, 4, 5]. Five year survival rate of GCA patients with distant metastasis is about 2-12% [1]. Thus, targeting metastasis of GCA is a key therapeutic approach for improving survival rate of GCA patients.

Establishing biomarkers and identifying target molecules of GCA metastasis are two important steps for diagnosis, prognosis and targeted therapy of metastasized GCA. Currently, down-regulation of expression of some genes, such as *RASSF2, RASSF6, FBXO32, GADD45A,* and *GADD45G*, by methylation has been found to associate with poor prognosis of GCA [6–9]. There are a number of proteins, such as c-Met, Her2, Pim-3, Myc, Sirt1, PLCE1, and CHOP proteins whose expression is correlated with progression of GCA [10–15]. In addition, expression or polymorphisms of microRNAs or long non-coding RNAs (lnc-RNAs) are also associated with progression of GCA [16–18]. Despite a number of biomarkers associated with poor prognosis or progression are established, few of metastatic biomarkers, especially metastatic driver proteins, in GCA have been identified. We have recently found that expression of an E3 ubiquitin ligase Nedd4-1 is significantly associated with metastasis of GCA and correlated with poor survival of GCA patients [19]. We also showed that Nedd4-1 functionally promotes gastric cancer cell migration and invasion, suggesting that Nedd4-1 is a driver protein for metastasis [19]. Further identification and characterization of novel biomarkers that are functionally associated with GCA metastasis are necessary for understanding of GCA progression, improving diagnosis, prognosis and therapy of GCA and developing anti-metastatic drugs for GCA targeted therapy.

CYR61 (or CCN1) is a member of CCN (Cysteine-rich 61(CYR61), connective tissue growth factor (CTGF), nephroblastoma overexpressed (NOV)) family that contains 6 highly homologous extracellular matrix (ECM) proteins involved in cell adhesion signaling [20]. CYR61 is a target gene product of the Hippo-YAP/TAZ transcription pathway [21–23]. The Hippo-YAP/TAZ pathway is known to promote tumor growth and metastasis [24–26]. CYR61 mediates the Hippo-YAP/TAZ signaling in activation of cancer cell adhesion, migration, metastasis, and angiogenesis [21, 27, 28]. We have shown that expression of CYR61 is up-regulated by geranylgeranylation signaling that activates the YAP/TAZ-mediated cell migration in the triple negative (ER-/PR-/Her2-) breast cancer MDA-MB-231 cells [21]. In addition, expression of CYR61 is associated with poor prognosis in breast cancer, ovarian cancer and esophageal squamous cell carcinoma [29–31]. These studies lead us to test CYR61 as a metastatic biomarker in GCA.

In this report, we have detected expression of CYR61 in tumor samples from 214 GCA cases by immunohistochemistry (IHC) staining using tissue microarray assay (TMA). Statistical analysis of the data has shown that CYR61 expression is positively correlated with metastasis of GCA and inversely associated with cumulative survival of GCA patients. Down-regulation of CYR61 by expression of the CYR61 shRNA in gastric cancer AGS cells impairs the cell migration and invasion, suggesting a driver role of CYR61 in metastasis. The results have demonstrated that CYR61 is a biomarker for prediction of poor prognosis of GCA and a potential molecular target for anti-metastatic therapy of GCA.

## RESULTS

### CYR61 is overexpressed in GCA tumor tissues and its expression is inversely correlated with cumulative survival of GCA patients

**Figure 1:**
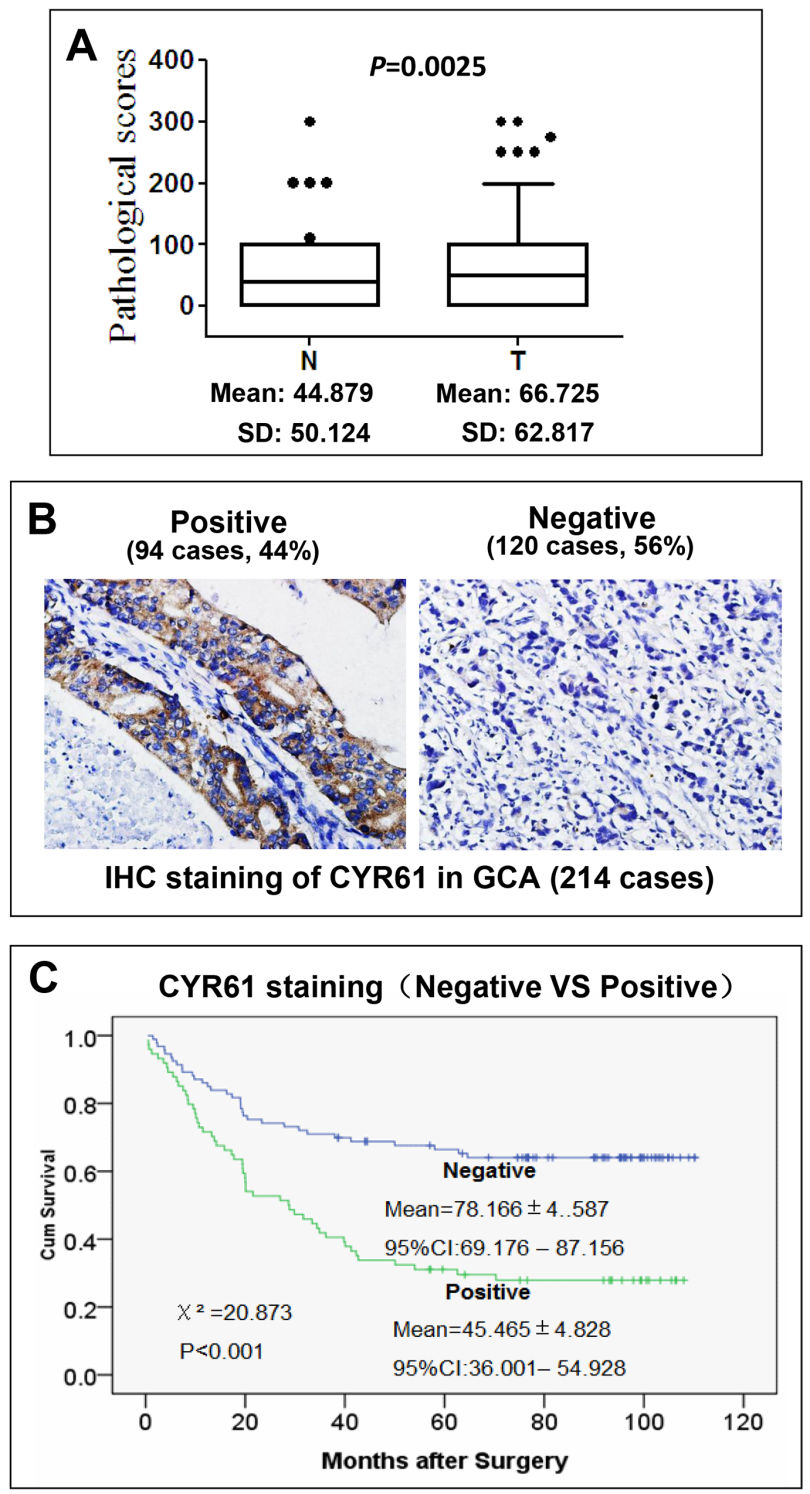
CYR61 is overexpressed in GCA and the overexpression is inversely correlated with cumulative survival of GCA patients **A.** Expression of CYR61 in GCA tumor tissue is significantly higher than in their adjacent normal gastric cardia tissue. The score distribution of IHC staining of CYR61 in both GCA tumors (T) and their adjacent normal tissue (N) is shown in the box plots. The black dots at top are scores outside of the box plots. The mean scores and standard deviation (SD) are shown at bottom of the box plots. **B.** IHC staining of CYR61 in GCA tumor samples. **C.** Kaplan-Meier survival curve of the CYR61 positive and negative GCA patients. The Chi-square and *p* values from Mantel-Cox test and the mean survival for the CYR61 negative and positive GCA are shown in the figure. The *p* value was calculated from Mantel-Cox test.

We detected CYR61 expression in both the GCA tumor tissues and the adjacent normal tissues from 214 GCA cases by IHC staining using tissue microarray assay (TMA). As shown Figure 1A, the average IHC staining score of CYR61 in the GCA tumor tissue is 66.725 with a standard deviation of 62.817 while in the normal tissue is 44.879 with a standard deviation of 50.124 (*p* = 0.0025), indicating that expression of CYR61 in the GCA tumor tissue is significantly higher than in the normal tissue. We further examined whether expression of CYR61 is associated with cumulative survival of GCA patients. The TMA assay has shown that 94 of the 214 GCA tumors (44%) were strongly stained with anti-CYR61, and 120 tumor samples (56%) had weak or no staining (Figure 1B), indicating that CYR61 is overexpressed in GCA with a high frequency.

To assess the role of CYR61 in prognosis, we determined association of CYR61 expression with cumulative survival of the post-surgery GCA patients by statistical analysis. As shown in the Kaplan-Meier survival graph (Figure 1C), the patients with the CYR61 negative tumor had average cumulative survival of 78.166 ± 4.587 months during the follow-up. On the other hand, the CYR61 positive patients had average cumulative survival of 45.465 ± 4.828 months (Figure 1C). Difference between the CYR61 positive and negative patient's cumulative survival is determined by log-rank test (Figure 1C). The chi-square value is 20.873 with *p*<0.001, indicating that cumulative survival between the CYR61 positive and the negative patients is dramatically different and that CYR61 expression is significantly inversely associated with post-surgery survival of GCA.

Using multivariate Cox regression model, we analyzed association of clinicopathological characteristics and CYR61 expression with AGC cumulative survival. Three variants, including TNM stage (*p*=0.049), N-categories (*p*=0.025), and CYR61 expression (*p*=0.001), are significantly inversely associated with cumulative survival of GCA (Table 1). Other variants, such as T category, differentiation and tumor size, are not significantly associated with cumulative survival. Among the three variants inversely associated with cumulative survival, CYR61 expression is the most significant one (*p*=0.001). The hazard ratio of CYR61 that is inversely correlated with cumulative survival equals to 0.461 with 95% CI of 0.286-0.742, which is comparable to the hazard ratio of N-category (HR=0.364, 95% CI=0.150-0.882) or TMN stage (HR=0.617, 95% CI=0.382-0.998). These results have demonstrated that CYR61 expression is an important clinical index for prediction of poor prognosis of GCA. Since TNM stage and N category are pathological indexes for metastasis, these data strongly suggest that metastasis is the cause for GCA mortality and that CYR61 is involved in metastasis of GCA.

**Table 1:** Multivariate Cox regression analysis of correlation of clinicopathological characteristics and CYR61 with AGC cumulative survival

Category	Hazard ratio (95%CI)	*P*
TMN stage	0.617 (0.382-0.998)	0.049
T category	0.513 (0.211-1.246)	0.140
N category	0.364 (0.150-0.882)	0.025
Differentiation	0.819 (0.521-1.288)	0.388
Tumor size	0.932 (0.317-2.739)	0.899
CYR61	0.461 (0.286-0.742)	0.001

### CYR61 is associated with metastasis of GCA

We further statistically analyzed correlation of CYR61 expression in pathological characteristics in GCA tumors. As shown in Table 2, CYR61 overexpression is highly associated with TNM stages (*p*=0.001), and significantly associated with N-category (*p*=0.052), but not with age (*p*=0.087), gender (*p*=0.435), tumor size (*p*=0.308), T-category (*p*=0.103) or grade (differentiation) (*p*=0.25). In TNM stages, CYR61 positive staining is observed in 53% of stage III/IV patients who had distant site metastasis or severe lymph node metastasis, compared to 30% of stage I/II who had no or minor lymph node metastasis. In N category, CYR61 positive staining is observed in 48% of N1-3 GCA patients (spread to regional lymph nodes), versus 36% for no lymph node spreading (*p*<0.001). These clinicopathological data suggest a strong association of CYR61 expression with GCA progression and metastasis.

**Table 2:** Association of CYR61 expression with pathological categories

Pathological category		Case numbers	CYR61 Positive	%	*P*
Age	<=60	108	42	38.9	0.087
	>60	106	52	49.11	
Gender	Male	157	70	44.6	0.435
	Female	57	24	42.1	
Tumor size	<=6cm	166	76	45.8	0.308
	>6cm	48	18	37.5	
T category	T1/T2	63	23	36.5	0.103
	T3/T4	151	71	47	
N category	N0	73	26	35.6	0.052
	N1-3	141	68	48.2	
Differentiation	Well/Mod	130	60	46.2	0.25
	Poor/undiff	84	34	40.5	
TNM stage	I/II	83	25	30.1	0.001
	III/IV	131	69	52.7	

### CYR61 plays a major role in gastric cancer cell migration and is required for EGF-stimulated gastric cancer cell migration and invasion

**Figure 2:**
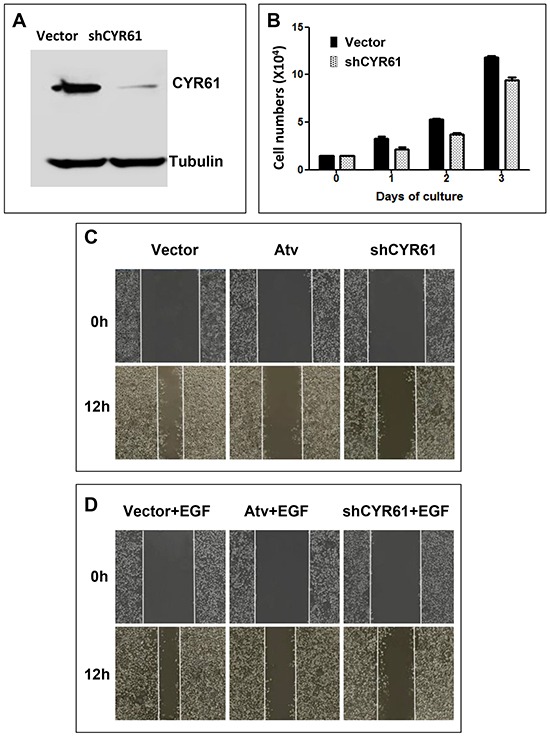

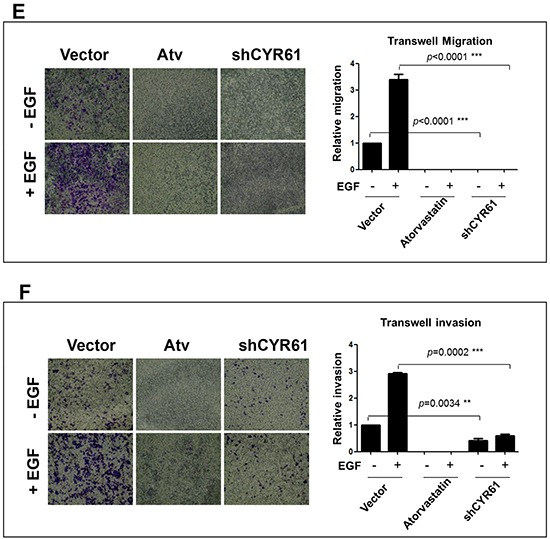
CYR61 is essential for cell migration and required for EGF-promoted migration and invasion in gastric cancer AGS cells **A.** Endogenous CYR61 in AGS cells was depleted by lentiviral vector-loaded shCYR61 for 48 hours and detected by immunoblotting with anti-CYR61 from the cell lysates. **B.** The effect of CYR61 knockdown on proliferation of AGS cells. **C-E.** The effect of CYR61 knockdown on migration of AGS cells. **C** and **D**, the wound healing assay. **E.** the transwell assay. **F.** The effect of CYR61 knockdown on invasion of AGS cells. The invasion was determined by the transwell matrigel invasion assay. The gastric cancer cell proliferation, migration and invasion assays were repeated three times. For quantification, the migrated or invasive cells were counted under a microscope from three randomly selected fields. Treatment with atorvastatin (Atv) (10 μM) was used as a positive control for inhibition of the Hippo-YAP/TAZ signaling. EGF (50 ng/ml) was used for stimulation of cell migration and invasion. ** *p* <0.01; *** *p*<0.001.

To address the driver role of CYR61 in metastasis, we attempted to determine if CYR61 directly regulates GCA metastatic properties by an *in vitro* assay. However, there is no GCA cell line available currently. Thus, we used AGS, which is a highly metastatic gastric cancer cell line [32], to mimic GCA cells in determination of the role of CYR61 in cell migration and invasion that are two important cellular processes in metastasis. Previous studies observed that ectopic expression of exogenous CYR61 enhanced migration and invasion of AGS cells [33], which may not conclude the cellular function of endogenous CYR61. To address the role of endogenous CYR61 in metastasis, we first established the CYR61-knockdown cell lines in AGS using lentiviral vector-loaded CYR61 shRNA. More than 90% of CYR61 was depleted in the sh-CYR61 cell lines (Figure 2A). Depletion of CYR61 caused a minor effect on cell proliferation (Figure 2B), suggesting that CYR61 plays an insignificant role in AGS cell proliferation. We then determined effect of CYR61 knockdown on cell migration and invasion. As shown in Figure 2C and 2D, depletion of CYR61 by the shRNA dramatically inhibited both basal and EGF-promoted cell migration using the wound healing assay. The inhibitory effect on cell migration is comparable with that treated with atorvastatin, an inhibitor of hydroxylmethylglutaryl co-enzyme A (HMG-CoA) reductase that blocks the Hippo-YAP/TAZ pathway in breast cancer cells [21, 34, 35]. In the transwell migration assays, both the basal and EGF-promoted cell migration was completely eliminated by knockdown of CYR61 (Figure 2E), which was the same as the effect of atorvastatin. Combining the data in Figure 2C, 2D and 2E, we conclude that CYR61 plays the major role in the Hippo-YAP/TAZ-mediated AGS cell migration signaling. In the transwell invasion assays, knockdown of CYR61 partially reduced the invasion capacity of AGS cells comparing to the effect of atorvastatin (about 50% of the effect of atorvastatin) (Figure 2F), suggesting that CYR61 plays a partial role in the Hippo-YAP/TAZ-mediated AGS cell invasion signaling.

Treatment of AGS cells with epidermal growth factor (EGF) induced a marked increase in AGS cell migration and invasion (Figures 2E and 2F), suggesting that EGFR signaling is involved in gastric cancer metastasis. Interestingly, both EGF stimulated migration and invasion of AGS cells were completely diminished by knockdown of CYR61 (Figures 2E and 2F), indicating that CYR61 is required for the migration or invasion signaling of EGFR in AGS cells.

**Figure 3:**
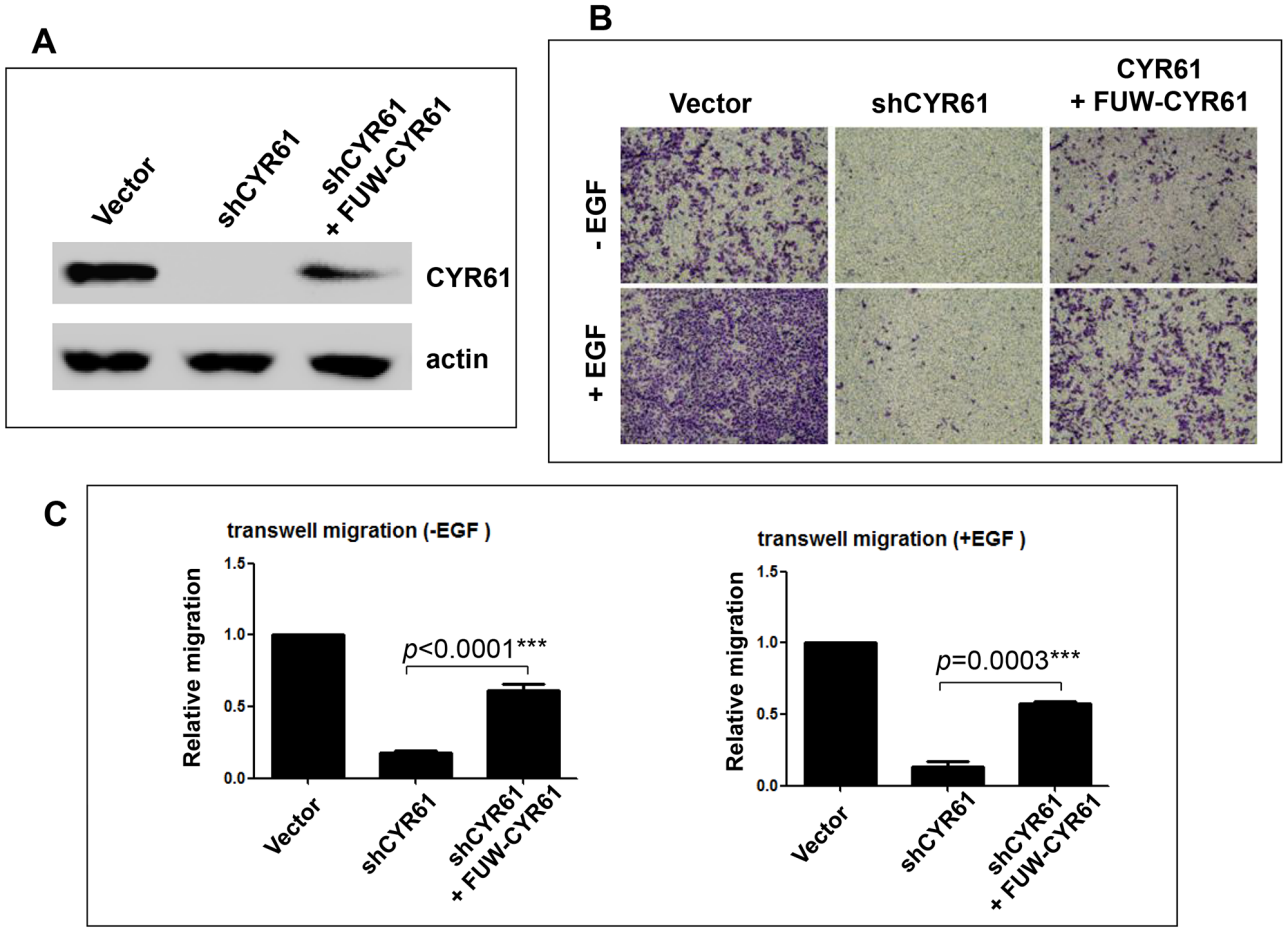
Re-expression of CYR61 rescues the inhibitory effect of CYR61 knockdown on cell migration **A.** The shCYR61 AGS cell line was infected by the lentiviral vector (FUW)-loaded CYR61 for 48 hours. The re-expressed CYR61 protein level was detected by immunoblotting. **B.** Migration of three different AGS cell lines was assessed by the transwell migration assay. **C.** For quantification, the dye crystal violet in the stained migrated cells was extracted with methanol and measured by absorbance at 540 nm. Data are from three independent experiments. EGF (50 ng/ml) was used for stimulation of cell migration. *** *p*<0.001.

To confirm the role of CYR61 in the gastric cancer cell migration, we re-expressed CYR61 using a lentiviral vector-loaded CYR61 expression system in the CYR61-knockdown AGS cell line (Figure 3A). The re-expression of CYR61 recovered about 50% of the protein level of CYR61 compared to the control cell line (Figure 3A). As expected, re-expression of CYR61 rescued impairment of both basal and EGF-stimulated cell migration caused by knockdown of CYR61 (Figures 3B and 3C). The recovery effect on the cell migration was about 50% (Figure 3C), corresponding to about 50% in recovered expression level of CYR61 (Figure 3A). These data clearly indicate that CYR61 is a driver protein required for the gastric cancer cell migration.

Taken together, the results from the IHC staining of GCA tumor tissues and the gastric cancer cell migration and invasion assays of the CYR61-knockdown AGS cells suggest that CYR61 is not only a clinical predictive index protein for poor prognosis of GCA but also a crucial driver protein in metastasis of GCA.

## DISCUSSION

Metastasis is the major cause contributing to the high mortality of GCA. However, few of biomarkers or driver proteins for GCA metastasis have been identified. Our studies demonstrated CYR61, a target gene product of the Hippo-YAP/TAZ transcription pathway, as a metastatic biomarker and driver of GCA. The IHC staining has shown that CYR61 is expressed in 44% of GCA tumors. CYR61 expression is inversely associated with cumulative survival (*p*<0.001) (Figure 1). More importantly, CYR61 expression is significantly correlated with N category (lymph node metastasis) (*p*=0.052) and TNM stage (remote metastasis) (*p*=0.001), but not with T category (*p*=0.103) or tumor size (*p*=0.308) (Table 2), suggesting that CYR61 functions specifically in GCA metastasis, but not tumor growth. Consistent with the data from clinicopathological analysis with IHC staining, knockdown of CYR61 in gastric cancer AGS cells severely impaired cell migration and invasion, the two key cellular processes in metastasis, but had a minor inhibitory effect on cell proliferation (Figures 2 and 3), suggesting that CYR61 is a driver for metastasis, but not for tumor growth. Our work has provided a new biomarker for metastasis and poor prognosis of GCA that might be used in clinical diagnosis and targeted therapy.

Previous studies have shown that promotion of angiogenesis is the major function of CYR61 in tumor progression [36, 37]. CYR61 promotes tumor angiogenesis through alphav/beta3 integrin-mediated cell adhesion and stimulates neo-vascularization [28]. Some studies also observed CYR61 activates the Wnt/Catenin, the Sonic Hedgehog (SHh), the MMP, the MAP kinase, the NF-κB and COX2 signaling pathways in tumor cells [33, 38, 39]. Expression of CYR61 is also associated with poor prognosis in breast, esophageal and ovarian cancers [29–31]. However, some studies observed a contradictory role of CYR61 in cancer progression and survival, particularly in lung cancer, and found that down-regulation of CYR61 is associated with poor prognosis [40–43]. One study reported that CYR61 expression is inversely correlated with gastric cancer progression through regulation of expression of MMP-7 [44]. These results suggest a complex role of CYR61, as an extracellular matrix protein, in regulation of cancer progression. Our studies clearly indicate that CYR61 functions in promoting metastasis. However, the mechanism by which CYR61 promoting metastasis in GCA currently is not known. It is possible that CYR61 activates NF-κB signaling to promote GCA metastasis, as shown in previous studies in gastric cancer metastasis [33]. We will look into this mechanism in future studies.

CYR61 is the known transcriptional target gene products of the Hippo-YAP/TAZ signaling [22]. The Hippo-YAP/TAZ pathway has been demonstrated to promote tumor growth and cancer cell migration and invasion [24]. Our previous studies have shown that geranylgeranylation signaling stimulates proliferation, migration and invasion of triple negative (ER-/PR-/Her-) breast cancer cells through activation of the Hippo-YAP/TAZ pathway and expression of CYR61, but not in ER+ breast cancer cell lines [21], suggesting that the Hippo-YAP/TAZ signaling is important for breast cancer progression. Thus, it is likely that Hippo-YAP/TAZ signaling pathway also plays an important role in GCA progression and metastasis. It is necessary to further investigate the role of the Hippo-YAP/TAZ pathway in GCA and develop the Hippo-YAP/TAZ pathway as a targeting pathway for GCA therapy.

## MATERIALS AND METHODS

### Materials

IHC staining S-P kit (KIT-9710) was purchased from MAIXIN Biology Corporation. Atorvastatin calcium was purchased from WuXi Sigma. Transwell dishes were purchased from Corning Inc. Matrigel was purchased from BD Biosciences. The gastric cancer cell line AGS was purchased from ATCC. Anti-CYR61 (SC-13100) was purchased from Santa Cruz, anti-Tubulin (G436) from Bioworld. The CYR61 shRNA oligos were synthesized by ShengGong Company.

### Human GCA tissue specimens and patient information

Human GCA tissue specimens were preserved in the Gastric Cancer Tissue Bank at Department of Oncology, Changzheng Hospital (Shanghai, China). Tissue microarrays containing 214 cases of primary gastric cardia adenocarcinoma from curative resection were used for detection of CYR61 expression. All of the tissue specimens for this study were obtained with patient informed consent, and the use of these GCA specimens was approved by the Changzheng and Changhai Hospital Institutional Review Board.

Clinicopathological information of the 214 GCA tumor samples has been published in our previous report [19] (also shown in Table 2). Briefly, in this GCA patient population, about three quarters of the patients were male. More than three quarters of the tumors were equal to or smaller than 6 centimeters. About two thirds of the patients were diagnosed with the tumors highly invaded (T3/T4) or metastasized to lymph nodes (N1-3 categories) or at advanced TNM stages (stage III/IV).

The post-surgery survival rate of the GCA patients was followed and censored [19]. Overall mean survival time of the patients is 64.036 ± 3.554 months (95% confidential interval (CI): 57.070 – 71.002). Although tumor sizes (<= 6 cm vs > 6 cm), invasive degrees (T1/T2 vs T3/T4), lymph node metastasis (N0 vs N1-3), differentiation grades (well/moderate vs poor/undifferentiated) and TNM stages (TNM I/II vs TNM III/IV) are all significantly and inversely associated with patient cumulative survival (*p*=0.003 or <0.001), the chi-square values from log-rank test indicate that the TNM-stage (χ^2^ = 50.396) is the most significant one inversely associated with cumulative survival, followed by N category (χ^2^ = 38.832) and T category (χ^2^ = 27.661) [19]. These data suggest that metastasis is the major factor associated with mortality of the GCA patients.

### Immunohistochemistry (IHC) staining

Standard procedure was performed to determine the level of CYR61 expression in the GCA tumor samples. Briefly, 4 μm sections of paraffin-embedded GCA tissue microarrays were de-paraffinized and rehydrated in xylene and alcohol bath solution. Antigen unmasking was performed by pretreatment of the slides in 0.01 M citrate buffer (pH 6.0) at 98°C for 5 min using a microwave oven. The slides were then cooled to room temperature. Endogenous peroxidase was eliminated by incubating the slides in 3% hydrogen peroxide for 10 min. After washed in 10 mM PBS (pH 7.4), the sections were incubated with normal goat serum at room temperature for 10 min, followed by incubation with anti-CYR61 antibody (dilution: 1:100) at 4°C overnight. An IHC staining S-P kit (KIT-9710; MAIXIN Biology Corporation, Fuzhou, China) was used to visualize antibody binding on the slides. Counterstaining was performed with hematoxylin. The IHC staining of CYR61 in these specimens was evaluated by two individuals under an Olympus CX31 microscope (Olympus, Center Valley, PA).

### Cell culture and knockdown of CYR61 by lentiviral vector-loaded shRNA in gastric cancer cells

HEK293T cells were cultured and maintained in DMEM (Hyclone) supplemented with 10% FBS at 37°C, 5% CO_2_. The gastric cancer AGS cells were grown in F12K (Boster bio) supplemented with 10% fetal bovine serum (Excell bio), 100 U/mL penicillin, and 100 mg/mL streptomycin in 5% CO2 at 37°C. In order to knockdown CYR61 (The targeting sequence: 5′-TTGAGGAGCATT AAGGTATTT-3′), we cloned CYR61 shRNA oligos (Forward oligo: 5′-CCGGTTGAGGAGCATTAAGGTA TTTCTCGAGAAAACCTTAATGCTCCTCATTTTTG-3′; Reverse oligo:5′-AATTCAAAAATTGAGGAG CATTAAGGTATTTCTCGAGAAATACCTTAATGCTCCTCAA-3′) into the shRNA cloning vector pLKO.1-TRC. The oligos were inserted into the AgeI/EcoRI sites of the vector. Lentiviral particle packaging was performed as following: HEK293T cells (1×10^6^) were plated in a 35 mm tissue culture plate overnight. The lentiviral shRNA plasmid (pLKO.1-shCYR61) was co-transfected with psPAX2 (Addgene) and pMD2.G (Addgene) into HEK293T cells for 8 hrs. The medium containing viral particles was collected every 24 hours for three times after transfection. The medium was centrifuged at 1250 rpm for 5 min to remove cell debris and used for infecting the AGS cells. For infection, AGS cells (1×10^5^) were plated in a 35 mm tissue culture dish overnight. One ml lentiviral particle solution was used for infecting AGS cells in presence of 6 μg/ml polybrene. The infected cells were selected with puromycin. The effect of CYR61 knockdown was detected by immunoblotting the cell lysates with anti-CYR61.

### Preparation of cell lysates and immunoblotting

Culture medium was removed and the cells were washed with cold PBS and lysed in precooled mammalian cell lysis buffer (40 mM HEPES, pH 7.4, 1% Triton X-100, 100mM NaCl, 1mM EDTA, 25 mM Beta-Glycerolphosphate, 1mM Na-orthovanadate, 10 μg/ml Leupeptin and 10 μg/ml Aprotinin). The SDS-PAGE samples were prepared by addition of 5 × SDS sample buffer directly to the lysates, followed by vortex and denatured at 100°C for 5 min. Electrophoresis was run on 12% SDS-PAGE gels, and separated proteins were transferred onto Immobilon PVDF membranes (Millipore). The membranes were incubated with primary antibodies (anti-CYR61 or anti-tubulin) overnight at 4°C, followed by incubating with secondary antibodies for 2 h at room temperature. The protein bands were detected by the Western Lightning ECL Detection Kit (Beytime).

### Determination of cell proliferation, migration and invasion

#### Determination of cell proliferation

The control or the CYR61 knockdown AGS cells were cultured in F12K with 10% FBS at 37°C plus 5% CO2. In a 12 well culture plate, 2×10^4^ AGS cells were plated in each well. The cells were trypsinized and counted under a phase microscope with a hemocytometer every 24 hrs. The cell counting was repeated at least three times. The histogram was drawn by Prism5.

#### Cell migration assays

Cell migration was detected by two methods: the wound-healing assay and the transwell assay. **The wound healing assay**. The cells (3×10^5^) were seeded on 12-well plates for 16 hrs (about 80-90% confluence), and scraped with a 10 μl pipette tip to create a wounding zone. Culture medium was replaced with the medium plus EGF or/and atorvastatin. The wound healing was assessed by photography every 12 hrs. The experiments were repeated at least three times. **The transwell assay**. AGS cells were trypsinized and resuspended in serum-free F12K medium. The density of cells was adjusted to 2×10^5^ /ml. The suspended cells (4×10^4^ in 200 μl) were gently added to the upper compartment of Transwell (Corning). F12K medium with 10% FBS or EGF (0.5 ml) was added to the lower compartment. After incubation for 24 hrs, the cells on upper side of the chamber were carefully removed, the cells migrated to bottom side of the chamber were fixed with 4% paraformaldehyde for 30 min and stained with 0.1% crystal violet solution. The stained cells were washed with PBS three times and visualized by photography. The migration experiments were repeated three times. For quantification, the cells were counted under a microscope from three randomly selected fields, or the dye crystal violet in the stained cells was extracted with methanol and measured by absorbance at 540 nm.

#### Cell invasion assay

Matrigel was melted at 4°C overnight. The Transwell top chamber membrane was coated with 40 μl of 0.125 mg/ml matrigel in F12K medium and incubated at 37°C for 3 to 5 hrs before use. AGS cells were trypsinized and washed with PBS two times and resuspended in serum-free F12K medium at a density of 2×10^5^ cells/ml. The cells (200 μl in serum-free F12K medium) were added to the top of the matrigel layer. The bottom chamber of the Transwell was filled with 500 μl of F12K medium plus 10% FBS or EGF. The cell invasion was carried out at 37°C and 5% CO_2_ for 24 hrs. After the invasion was done, the cells on upper side of the chamber were removed by cotton balls. The cells on the bottom side (invaded cells) were fixed with 4% paraformaldehyde at 25°C for 30 min and stained with 0.1% crystal violet solution at 25°C for 20 min. The stained cells were washed with PBS three times and observed and photographed under a microscope. For quantification, the cells were counted under a microscope from three randomly selected fields.

### Statistical analysis

The association between clinicopathological variables and CYR61 expression was tested using chi-square test. Kaplan-Meier survival analysis was conducted from post-surgery to death, stratified by CYR61 expression status, TNM stage, T category, N category, tumor size, and differentiation, respectively. The multivariate Cox proportional hazard model was used to determine the association between CYR61 status and survival, controlling for tumor size, differentiation, T category, N category and TNM stage. Hazard ratio (HR) and 95% confidence interval (95% CI) were calculated for each factor. Interactions were also tested for each pair of factors. *P*-value < 0.05 was considered to be statistically significant, and all analyses were conducted using IBM SPSS software (IBM Corp, Armonk, NY).

## References

[1] Buas MF, Vaughan TL (2013). Epidemiology and risk factors for gastroesophageal junction tumors: understanding the rising incidence of this disease. Semin Radiat Oncol.

[2] Ohno S, Tomisaki S, Oiwa H, Sakaguchi Y, Ichiyoshi Y, Maehara Y, Sugimachi K (1995). Clinicopathologic characteristics outcome of adenocarcinoma of the human gastric cardia in comparison with carcinoma of other regions of the stomach. J Am Coll Surg.

[3] Rüdiger Siewert J, Feith M, Werner M, Stein HJ (2000). Adenocarcinoma of the esophagogastric junction: results of surgical therapy based on anatomical/topographic classification in 1,002 consecutive patients. Ann Surg.

[4] Papachristou DN, Fortner JG (1980). Adenocarcinoma of the gastric cardia. The choice of gastrectomy. Ann Surg.

[5] Nakane Y, Okamura S, Boku T, Okusa T, Tanaka K, Hioki K (1993). Prognostic differences of adenocarcinoma arising from the cardia the upper third of the stomach. Am Surg.

[6] Guo W, Dong Z, Guo Y, Shen S, Guo X, Kuang G, Yang Z Decreased expression and frequent promoter hypermethylation of RASSF2 and RASSF6 correlate with malignant progression and poor prognosis of gastric cardia adenocarcinoma. Mol Carcinog.

[7] Guo W, Zhang M, Guo Y, Shen S, Guo X, Dong Z (2015). FBXO32, a new TGF-β/Smad signaling pathway target gene, is epigenetically inactivated in gastric cardia adenocarcinoma. Neoplasma.

[8] Guo W, Dong Z, Guo Y, Chen Z, Kuang G, Yang Z (2013). Methylation-mediated repression of GADD45A and GADD45G expression in gastric cardia adenocarcinoma. Int J Cancer.

[9] Guo W, Dong Z, Guo Y, Lin X, Chen Z, Kuang G, Yang Z (2013). Aberrant methylation and loss expression of RKIP is associated with tumor progression and poor prognosis in gastric cardia adenocarcinoma. Clin Exp Metastasis.

[10] Chi F, Fu D, Zhang X, Lv Z, Wang Z (2012). Expression of the c-Met proto-oncogene and Integrin α5β1 in human gastric cardia adenocarcinoma. Biosci Biotechnol Biochem.

[11] Katai H, Ishida M, Yamashita H, Ohashi M, Morita S, Katayama H, Fukagawa T, Kushima R (2014). HER2 expression in carcinomas of the true cardia (Siewert type II esophagogastric junction carcinoma). World J Surg.

[12] Lou L, Wang Y1, Cui J, Yan X, Xue L, Li Y (2014). Differential expression of Pim-3, c-Myc, and p-p27 proteins in adenocarcinomas of the gastric cardia and distal stomach. Tumour Biol.

[13] Feng AN, Zhang LH, Fan XS, Huang Q, Ye Q, Wu HY, Yang J (2011). Expression of SIRT1 in gastric cardiac cancer and its clinicopathologic significance. Int J Surg Pathol.

[14] Li WQ, Hu N, Burton VH, Yang HH, Su H, Conway CM, Wang L, Wang C, Ding T, Xu Y, Giffen C, Abnet CC, Goldstein AM, Hewitt SM, Taylor PR (2014). PLCE1 mRNA and protein expression and survival of patients with esophageal squamous cell carcinoma and gastric adenocarcinoma. Cancer Epidemiol Biomarkers Prev.

[15] Zhu XJ, Gao SG, Li SQ, Shi ZG, Ma ZK, Zhu SS, Feng XS (2015). Down-regulation of C/EBP homologous protein (CHOP) expression in gastric cardia adenocarcinoma: Their relationship with clinicopathological parameters and prognostic significance. Clin Res Hepatol Gastroenterol.

[16] Wang S, Tao G, Wu D, Zhu H, Gao Y, Tan Y, Wang M, Gong W, Zhou Y, Zhou J, Zhang Z (2013). A functional polymorphism in MIR196A2 is associated with risk and prognosis of gastric cancer. Mol Carcinog.

[17] Wang Y, Feng X, Jia R, Liu G, Zhang M, Fan D, Gao S (2014). Microarray expression profile analysis of long non-coding RNAs of advanced stage human gastric cardia adenocarcinoma. Mol Genet Genomics.

[18] Guo W, Dong Z, Bai Y, Guo Y, Shen S, Kuang G, Xu J (2015). Associations between polymorphisms of HOTAIR and risk of gastric cardia adenocarcinoma in a population of north China. Tumour Biol.

[19] Sun A, Yu G, Dou X, Yan X, Yang W, Lin Q (2014). Nedd4-1 is an exceptional prognostic biomarker for gastric cardia adenocarcinoma and functionally associated with metastasis. Mol Cancer.

[20] Brigstock DR (1999). The connective tissue growth factor/cysteine-rich 61/nephroblastoma overexpressed (CCN) family. Endocr Rev.

[21] Mi W, Lin Q, Childress C, Sudol M, Robishaw J, Berlot CH, Shabahang M, Yang W (2015). Geranylgeranylation signals to the Hippo pathway for breast cancer cell proliferation and migration. Oncogene.

[22] Pobbati AV, Hong W (2013). Emerging roles of TEAD transcription factors and its coactivators in cancers. Cancer Biol Ther.

[23] Zhang Y, Xia H, Ge X, Chen Q, Yuan D, Chen Q, Leng W, Chen L, Tang Q, Bi F (2014). CD44 acts through RhoA to regulate YAP signaling. Cell Signal.

[24] Yu FX, Zhao B, Guan KL (2015). Hippo Pathway in Organ Size Control, Tissue Homeostasis, and Cancer. Cell.

[25] Lamar JM, Stern P, Liu H, Schindler JW, Jiang ZG, Hynes RO (2012). The Hippo pathway target, YAP, promotes metastasis through its TEAD-interaction domain. Proc Natl Acad Sci U S A.

[26] Nallet-Staub F, Marsaud V, Li L, Gilbert C, Dodier S, Bataille V, Sudol M, Herlyn M, Mauviel A (2014). Pro-invasive activity of the Hippo pathway effectors YAP and TAZ in cutaneous melanoma. J Invest Dermatol.

[27] Bleau AM, Planque N, Perbal B (2005). CCN proteins and cancer: two to tango. Front Biosci.

[28] Babic AM, Kireeva ML, Kolesnikova TV, Lau LF (1998). CYR61, a product of a growth factor-inducible immediate early gene, promotes angiogenesis and tumor growth. Proc Natl Acad Sci U S A.

[29] Saglam O, Dai F, Husain S, Zhan Y, Toruner G, Haines GK (2014). 3rd Matricellular protein CCN1 (CYR61) expression is associated with high-grade ductal carcinoma in situ. Hum Pathol.

[30] Xie JJ, Xu LY, Wu ZY, Li LY, Xu XE, Wu JY, Huang Q, Li EM (2011). Expression of cysteine-rich 61 is correlated with poor prognosis in patients with esophageal squamous cell carcinoma. Eur J Surg Oncol.

[31] Shen H, Cai M, Zhao S, Wang H, Li M, Yao S, Jiang N (2014). CYR61 overexpression associated with the development and poor prognosis of ovarian carcinoma. Med Oncol.

[32] Li X, Zhang Y, Cao S, Chen X, Lu Y, Jin H, Sun S, Chen B, Liu J, Ding J, Wu K, Fan D (2009). Reduction of TIP30 correlates with poor prognosis of gastric cancer patients and its restoration drastically inhibits tumor growth and metastasis. Int J Cancer.

[33] Lin MT, Zuon CY, Chang CC, Chen ST, Chen CP, Lin BR, Wang MY, Jeng YM, Chang KJ, Lee PH, Chen WJ, Kuo ML (2005). Cyr61 induces gastric cancer cell motility/invasion via activation of the integrin/nuclear factor-kappaB/cyclooxygenase-2 signaling pathway. Clin Cancer Res.

[34] Wang Z, Wu Y, Wang H, Zhang Y, Mei L, Fang X, Zhang X, Zhang F, Chen H, Liu Y, Jiang Y, Sun S, Zheng Y, Li N, Huang L (2014). Interplay of mevalonate and Hippo pathways regulates RHAMM transcription via YAP to modulate breast cancer cell motility. Proc Natl Acad Sci U S A.

[35] Sorrentino G, Ruggeri N, Specchia V, Cordenonsi M, Mano M, Dupont S, Manfrin A, Ingallina E, Sommaggio R, Piazza S, Rosato A, Piccolo S, Del Sal G (2014). Metabolic control of YAP and TAZ by the mevalonate pathway. Nat Cell Biol.

[36] Lau LF (2011). CCN1/CYR61: the very model of a modern matricellular protein. Cell Mol Life Sci.

[37] Kuonen F, Secondini C, Rüegg C (2012). Molecular pathways: emerging pathways mediating growth, invasion, and metastasis of tumors progressing in an irradiated microenvironment. Clin Cancer Res.

[38] Li ZQ, Ding W, Sun SJ, Li J, Pan J, Zhao C, Wu WR, Si WK (2012). Cyr61/CCN1 is regulated by Wnt/β-catenin signaling and plays an important role in the progression of hepatocellular carcinoma. PLoS One.

[39] Haque I, De A, Majumder M, Mehta S, McGregor D, Banerjee SK, Van Veldhuizen P, Banerjee S (2012). The matricellular protein CCN1/Cyr61 is a critical regulator of Sonic Hedgehog in pancreatic carcinogenesis. J Biol Chem.

[40] van Ginkel PR, Gee RL, Shearer RL, Subramanian L, Walker TM, Albert DM, Meisner LF, Varnum BC, Polans AS (2004). Expression of the receptor tyrosine kinase Axl promotes ocular melanoma cell survival. Cancer Res.

[41] Chien W, Kumagai T, Miller CW, Desmond JC, Frank JM, Said JW, Koeffler HP (2004). Cyr61 suppresses growth of human endometrial cancer cells. J Biol Chem.

[42] Tong X, O'Kelly J, Xie D, Mori A, Lemp N, McKenna R, Miller CW, Koeffler HP (2004). Cyr61 suppresses the growth of non-small-cell lung cancer cells via the beta-catenin-c-myc-p53 pathway. Oncogene.

[43] Mori A, Desmond JC, Komatsu N, O'Kelly J, Miller CW, Legaspi R, Marchevsky AM, McKenna RJ, Koeffler HP (2007). CYR61: a new measure of lung cancer outcome. Cancer Invest.

[44] Maeta N, Osaki M, Shomori K, Inaba A, Kidani K, Ikeguchi M, Ito H (2007). CYR61 downregulation correlates with tumor progression by promoting MMP-7 expression in human gastric carcinoma. Oncology.

